# Endoscopic interventional therapies for tracheoesophageal fistulas in children: A systematic review

**DOI:** 10.3389/fped.2023.1121803

**Published:** 2023-02-22

**Authors:** Yaozheng Ling, Bingyue Sun, Junhui Li, Li Ma, Deli Li, Guo Yin, Fanzheng Meng, Man Gao

**Affiliations:** ^1^ Department of Pediatrics, The First Hospital of Jilin University, Changchun, China; ^2^ Medical Insurance Office, The First Hospital of Jilin University, Changchun, China; ^3^ Center for Pathogen Biology and Infectious Diseases, The First Hospital of Jilin University, Changchun, China

**Keywords:** tracheoesophageal fistula, endoscopic interventional therapies, treatment, success rate, children

## Abstract

**Methods:**

An electronic literature search was performed using the keywords “tracheoesophageal fistula,” “endoscopic,” and “children” in the four major medical databases (Ovid, Embase, PubMed, and Web of Science) right from inception to September 2022. All English language articles describing the endoscopic interventional therapies of TEF in children were reviewed. Two independent researchers screened eligible articles at the title and abstract level. Full texts of potentially relevant articles were then screened again, and reference lists were screened manually to identify additional studies. Relevant data were extracted and analyzed. A synthesis of the relevant data was presented in descriptive form because of the heterogeneity of the included articles. The Chi-Squared test was used with a significance level of 5% (*P *< 0.05).

**Results:**

Among the 1,167 retrieved papers, a total of 46 studies describing 170 TEF patients with an age range of 0.3–175 months were included, including 11 cases of acquired tracheoesophageal fistula, 144 cases of recurrent tracheoesophageal fistula, and 15 cases of congenital tracheoesophageal fistula (H-type TEF). A total of 119 out of 170 fistulas were successfully blocked *via* endoscopic techniques with an overall success rate of 70.0%, while 48 fistulas failed to close by endoscopic interventions, following which the procedure was converted to open surgery. No obviously severe intraoperative/postoperative complications occurred during the follow-up period, but only a mild esophageal stricture was noticed in six patients and grade II tracheal stenosis in one patient. Two patients died from causes unrelated to endoscopic procedures, with a mortality rate of approximately 1.2%. A comparative assessment of different endoscopic interventional techniques for TEF that detected endotracheal stenting was performed in six patients and one fistula was successfully blocked (16.7%). De-epithelialization alone was performed in 65 patients and the fistula healed in 47 of them (72.3%), with the mean number of successful treatments required being 2.3 times. Chemical sealant injection was administered in 33 patients and success was achieved in 21 (63.6%). The average requirement for endoscopic procedures was 1.5 times. De-epithelialization, in combination with chemical sealant injection, was performed in 62 patients, achieving the highest success rate of 77.4% (48 patients). Other treatment methods were performed in four patients and successfully treatment outcomes were reported in two of them (50.0%). The mean number of successful treatments required was four times, and a treatment was converted to surgery in one patient (25.0%). An assessment of different TEF types showed that 9 out of 15 congenital TEFs, 7 out of 11 acquired TEFs, and 103 out of 144 recurrent TEFs were successfully occluded. A comparison of the success rate across multiple groups showed a significant difference with a score of *P *< 0.05, while there was no significant difference in the success rate of different TEF-type groups (*P *> 0.05).

**Conclusion:**

Endoscopic intervention is currently a preferred treatment modality for children with TEF because of its less-invasive nature, less complications, and high success rate. Among all interventional techniques, de-epithelialization, in combination with chemical sealant, has a higher success rate than other techniques. However, due to the limited number of cases reported for implementing many kinds of techniques, an ideal endoscopic interventional technique has yet to be devised, often necessitating more treatment applications and close follow-up.

## Introduction

Tracheoesophageal fistula (TEF) is a congenital or acquired pathological entity characterized by the presence of an abnormal communication between the posterior aspect of the trachea and the anterior wall of the esophagus (1, 2). Congenital TEF is a rare congenital respiratory anomaly resulting from a developmental disruption occurring within the 4th to 6th week of gestation, when separation of the trachea and esophagus occurs by folding of the embryogenic foregut (2–4). This TEF is usually classified into the following five types according to the location of the atresia and the presence of any fistula associated to the trachea (5): Type A with isolated esophageal atresia (EA) without TEF, Type B with proximal fistula and distal esophageal atresia, Type C with proximal esophageal atresia and distal fistula, Type D with proximal and distal fistula, and Type E is a “H-type” fistula but without esophageal atresia. Type C is the primary type of congenital TEF, accounting for 78%–90% of cases, while H-type TEF accounts for only 4% of cases (2, 6–8). Acquired TEF is divided into acquired malignant tracheoesophageal fistulas and acquired non-malignant or benign tracheoesophageal fistulas, which are the major type in children caused by accidental swallowing of foreign bodies (button batteries, metals, corrosive liquids, etc.), trauma, medical trauma, burns, etc. (9, 10). A recurrent TEF after EA/TEF repair is also similar to a congenital H-type TEF in terms of its physiological anatomy due to a previous surgical remodeling of the esophagus. With the development of surgical and thoracoscopic techniques and improvements in anesthesia and perioperative management, an increasing number EA/TEFs can be successfully treated. However, the significant recurrence and mortality rates associated with surgery should not be underestimated, and recurrent TEF is reported in 3% to 20% of infants following the repair of EA/TEF (9, 11, 12).

With granulation proliferation and crusting around the fistula after previous surgical repair, there is a greater risk of damage to the surrounding tissues, nerves, and blood vessels during reopening or another thoracoscopic surgical repair of recurrent TEF. Also, due to the sustained chemical damage to the tissues around the tracheoesophageal fistula occurring on account of accidental factors, such as button battery ingestion, the tissue around the fistula becomes so edematous and necrotic that secondary tears easily occur during another surgical repair (13). Therefore, surgical repair for acquired TEF and recurrent TEF is not an easy and suitable option. In light of these factors, endoscopic interventions are increasingly becoming the first choice of treatment for these types of TEF in children because of their flexible equipment, minimal trauma, high success rate of closure, and repeatability.

In this study, we performed a systematic review of the published endoscopic interventional therapies for TEF in children and attempted to provide a guidance for pediatricians on the choice of endoscopic interventions to seal TEF in children.

## Materials and methods

The study was carried out according to the Preferred Items for Reporting of Systematic Reviews and Meta-Analyses (PRISMA) guidelines (14). The study protocol was not registered.

### Search strategy

An electronic literature search was performed using the keywords “tracheoesophageal fistula,” “endoscopic,” and “children” in the four major medical databases (Ovid, Embase, PubMed, and Web of Science) right from inception to September 2022. All English language articles describing the endoscopic interventional therapies of TEF in children were reviewed. Two independent researchers screened eligible articles at the title and abstract levels. Full texts of potentially relevant articles were then screened again, and reference lists were screened manually to identify additional studies. Relevant data were extracted and analyzed. A synthesis of the relevant data was presented in descriptive form due to the heterogeneity of the included articles.

### Inclusion and exclusion criteria of the literature

Inclusion criteria: (1) literature with diagnosed tracheoesophageal fistula cases; (2) literature detailing endoscopic interventional techniques for TEF; (3) literature reporting outcomes and prognosis after endoscopic treatment of children with TEF; (4) cases in children; (5) literature published in peer-reviewed publications; (6) literature in only the English language. Exclusion criteria: (1) surgical repair treatment; (2) non-pediatric cases; (3) those on whom no clear endoscopic treatment intervention technique was performed or those who were subjected to only endoscopic examination and evaluation or were provided assistance in treatment; (4) literature related only to postoperative TEF nursing care records or anesthesia technique; (5) literature related to animal research; (6) literature with missing or incomplete clinical data. Studies that fulfilled the criteria regardless of the different types (although always > 1) of endoscopic interventional therapy, the number of participants, and the outcome of treatment were eligible for inclusion in the review process.

### Literature screening process

**Figure 1:**
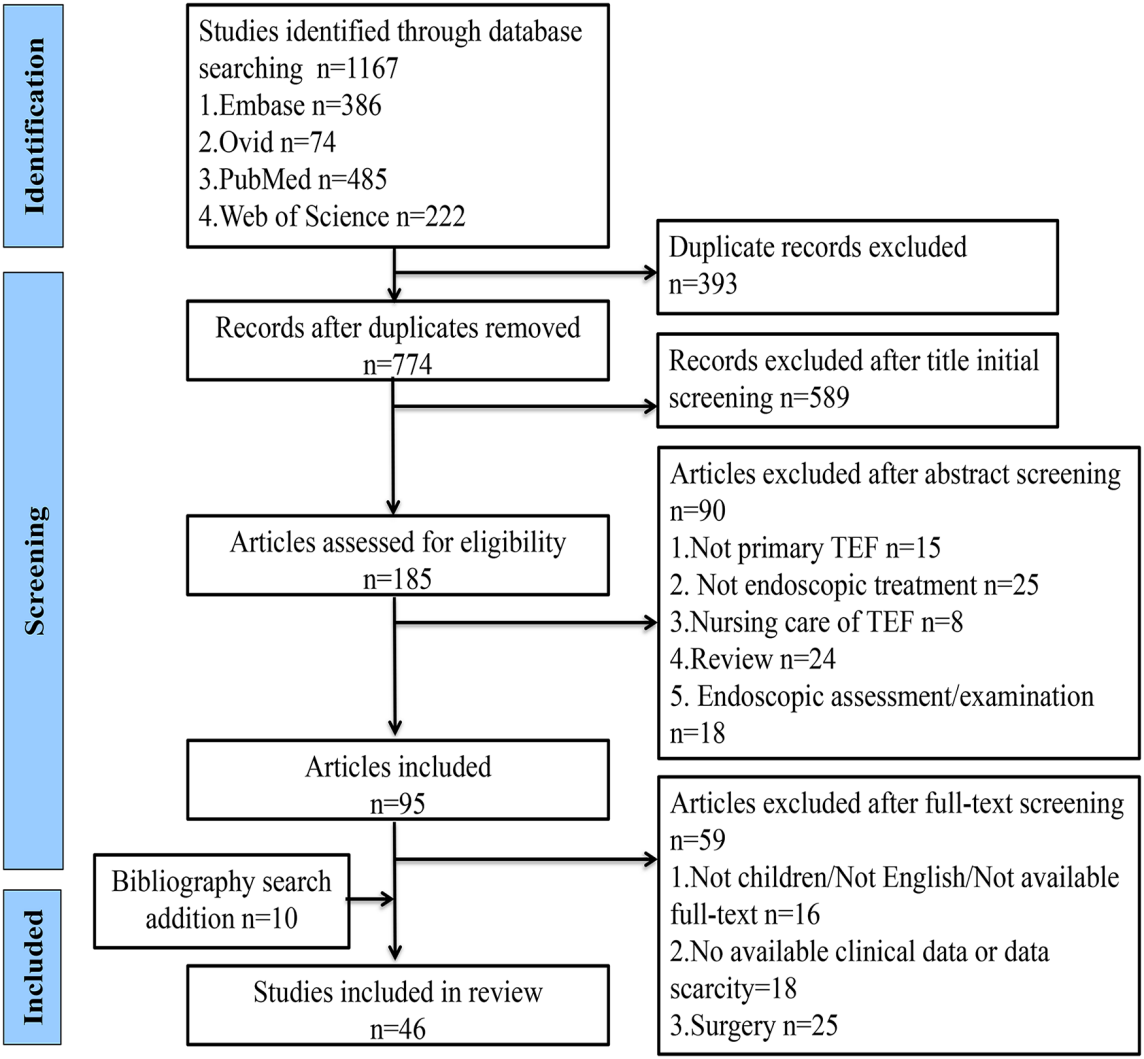
PRISMA flow diagram for systematic search and screening.

The search was conducted in the Ovid, Embase, PubMed, and Web of Science databases using the formula “tracheoesophageal fistula AND endoscopic AND children,” and the time frame for each database was set from inception to September 2022. The retrieved literature was simultaneously imported into EndNote software, and the duplicate literature from different databases was removed following the initial title and abstract screening to exclude literature that did not have tracheoesophageal fistula as a primary study, without endoscopic interventional procedures, related to nursing care, endoscopic evaluation, and examination, and reviews. The full text was read to exclude articles that were not about children, not in English, and not available in full text and also those with incomplete data, surgical repair, endoscopic-assisted treatment, and reviews. The included literature was further screened and analyzed to include additional cases from the bibliographic search according to the inclusion and exclusion criteria. Cases with unclear information would be further identified through email contact to the authors of the literature (Figure 1).

### Data extraction and statistical analysis

The literature was screened, extracted, and processed by two independent researchers according to the inclusion and exclusion criteria, and if any disagreements arose during the screening process, a third researcher would offer his or her opinion until consensus was reached. A numerical table was used to extract data, and if two papers reported duplicate information of the same patient, only those with complete outcome indicators were included. The following data were extracted from each included study: authors, time and type of study publication, number of reported cases, age, clinical presentations before bronchoscopic intervention, type of tracheoesophageal fistula, endoscopic treatment technique, and intraoperative or postoperative complications. Non-normal data were described as the median (min − max), while the counts were expressed as the number of cases and percentages. Comparisons of the success rate across groups were conducted using the Chi-Square test at a significance level of *P *< 0.05.

### Data presentation

The PRISMA statement for Reporting Systematic Reviews was used to conduct the review and report its results. The first part of this systematic review focused on the parameters that were related to assess the endoscopic interventional therapies of TEF. The findings were then organized according to the characteristics of the different endoscopic interventional techniques.

## Results

### Profile of cases from included literature

A total of 46 studies describing 170 cases of patients with tracheoesophageal fistula in the age range of 0.3–175 months were included in this review (9, 15–59), as shown in Supplementary Table S1. The literature screening course is shown in Figure 1. A total of 119 out of 170 TEFs were successfully blocked *via* endoscopic techniques and the overall success rate was 70.0%. A total of 48 cases (28.2%) of patients were converted to open surgery, while two patients (1.2%) died. During the follow-up period of 0.5–264 months, one patient had tracheal posterior membrane necrosis with final grade II tracheal stenosis and six patients occasionally developed mild esophageal stricture. The 170 children with TEF included 144 recurrent cases, 15 congenital (H-type) cases, and 11 acquired ones. The acquired TEFs were caused by button battery ingestion in five patients, accidental swallowing of a ballpoint pen and an unknown foreign body in one patient each, trauma in two, and mechanical ventilation in two. The preperformance symptoms include choking on feeding, recurrent coughing and respiratory infections, and so on.

### Comparative analysis of different endoscopic interventional techniques

As shown in Table 1, a total of six patients in an age range of 2–29 months were treated through isolated endotracheal stent placement without esophageal stents—five patients with covered metal biliary stents and one with a silicone stent. Of these six patients, three had the H-type TEF and three acquired TEF, and one of which (16.7%) was successfully closed in a single session. In another five patients, full closure could not be achieved, following which the treatment modality in four patients was converted to open surgery (66.7%), and in the other, it was finally obliterated by placing several endoclips after removing the stent. According to the profile of this endoscopic technique group, no death occurred and there were no obvious severe complications during the follow-up period of 2–25 months (Table 1).

**Table 1:** Published outcomes of different endoscopic interventional techniques for TEF.

Descriptive data	Stenting	De-epithelialization + sealant	De-epithelialization	Sealant	Others	*χ* ^2^	*p*-value
Number of patients	6	62	65	33	4	—	—
Age range at treatment in months	2–29	0.3–108	0.5–175	0.5–144	0.5–72	—	—
Mean number of treatments required	1.0	1.9	2.3	1.5^b^	4	—	—
Treatment failures converted to open surgery, *n* (%)	4 (66.7%)	13 (21.0%)	18 (27.7%)	12 (36.4%)	1	—	—
Length of follow-up in months	2–25	1–240	3–96	3–264	0.5–48	—	—
Successful rate, *n* (%)	1 (16.7%)^a^	48 (77.4%)^a^	47 (72.3%)	21 (63.6%)	2 (50.0%)	10.496	0.024
Number of deaths, *n* (%)	0	1 (1.6%)	0	0	1 (25.0%)	—	—
Type	3A + 1R + 2C	53R + 4C + 5A	54R + 11C	33R	3A + 1R	—	—
Notes	One patient’s condition was obliterated by several endoclips after failure in stenting	One patient died after the procedure because of causes unrelated to the endoscopic intervention	Tracheal posterior membrane necrosis occurred with final tracheal stenosis grade II in 1 patient		One patient died of fungal septicemia	—	—

A, acquired TEF; R, recurrent TEF; C, congenital TEF; TEF, tracheoesophageal fistula.

^a^ Stenting group and group of the combination of de-epithelialization and sealant have statistically significant differences in the success rate (P < 0.05).

^b^ Cases from Willetts et al. (53) are not included because the number of required treatments is not available.

A total of 65 patients were included in the de-epithelialization alone group in the age range of 0.5–175 months, of which in 47 patients—41 recurrent TEFs and 6 congenital TEFs—fistulas were successfully closed with an overall success rate of 72.3%. However, in 18 patients (27.7%), occlusion could not be achieved through bronchoscopic interventional treatment, following which the treatment was converted to open surgery. In addition, the average number of treatments required for achieving successful outcomes was approximately 2.3 times. During the follow-up period of 3–96 months, no deaths occurred; however, tracheal posterior membrane necrosis occurred in one patient after endoscopic repair and also final left grade II tracheal stenosis.

The age range of children in the chemical sealant blocking group was 0.5–144 months, and all 33 children had recurrent TEF, of which 21 were successfully blocked with an overall success rate of 63.6%. The average number of treatments required for producing successful outcomes was approximately 1.5 times. However, in 12 patients (36.4%), the treatment was converted to open surgery. During the follow-up period of 3–264 months, there were no obvious complications and deaths.

A total of 62 patients—53 recurrent TEFs, 4 congenital TEFs, and 5 acquired TEFs—were included in the de-epithelialization group, in combination with the chemical sealant group, with an age range of 0.3–108 months. Of these patients, successfully closure was achieved in 48 (77.4%), and the average number of successful treatments needed was approximately 1.2 times; 13 procedures (21.0%) were converted to open surgery. One patient (1.6%) died after the operation, but the cause was unrelated to the surgery.

Apart from the techniques mentioned above, several other bronchoscopic interventional techniques such as an insertion of the cardiac Amplatzer septal and a flexible endoscopic suturing device and endoscopic submucosal dissection were used in four patients. Two acquired TEFs could not be closed through the insertion of the cardiac Amplatzer septal, one patient was kept stable for 4 years and finally the treatment was converted to surgery, and one died after being critically ill with fungal septicemia. One case of acquired TEF was successfully closed by endoscopic submucosal dissection, combined with endoscopic clips, and a recurrent TEF case was successfully closed with the flexible endoscopic suturing device.

A comparison of the success rate across multiple endoscopic interventional technique groups showed significant differences with a *P *< 0.05 (Table 1, *P *= 0.024), and the combined de-epithelialization and sealant technique seemed to have the highest success rate (77.4%). However, a comparison of these different techniques in the two groups did not show any significant difference (*P *> 0.05), as also a comparison done in the stenting group and the combined de-epithelialization and sealant groups (*P *< 0.05).

### Comparative analysis of different types of TEF

As shown in Table 2, 103 cases of recurrent TEF were successfully blocked with a success rate of 71.5%, of which 41 were blocked by the de-epithelialization technique alone, 21 by sealant alone, 40 by a combination of the de-epithelialization and sealant techniques, and 1 by inserting the flexible endoscopic suturing device. Nine out of 15 cases (60%) of congenital TEF were successfully blocked, and of these, 6 out of 11 cases were blocked by the de-epithelialization technique and three out of four cases by the combination of the de-epithelialization and sealant techniques. Seven out of eleven cases (63.6%) of acquired TEF were successfully blocked: one by stenting, five by the combination of the de-epithelialization and sealant techniques, and one by endoscopic submucosal dissection combined with endoscopic clips. Many TEF cases usually require more than one endoscopic intervention, as indicated by Table 2. The mean number of treatments required for congenital TEF was 1.2, while 1.9 times and 2.2 times of procedures were required for acquired TEF and recurrent TEF, respectively. The comparison of the success rate across multiple groups of different TEF types did not show any significant difference, with a *P* > 0.05 (*P *= 0.568) (Table 2).

**Table 2:** Summary of results comparing different types of tracheoesophageal fistula.

Descriptive data	Congenital TEF (H-type)	Acquired TEF	Recurrent TEF	*χ* ^2^	*P*-value
Number of patients	15	11	144	—	—
Mean number of treatments required	1.2	1.9	2.2^a^	—	—
Successful patients, *n* (%)	9 (60.0%)	7 (63.6%)	103 (71.5%)	1.33	0.568
Endoscopic interventional techniques (*n*1 – *n*2)^b^	De-epithelialization 11-6, de-epithelialization + sealant 4-3	Stenting 3-1, de-epithelialization + sealant 5-5, Others 3-1	Stenting 3-0, de-epithelialization 54-41, sealant 33-21, de-epithelialization + sealant 53-40, others 1-1	—	—

TEF, tracheoesophageal fistula.

Note: When multiple techniques were used on one patient, the last technique described was used to deduce the final outcome.

^a^ Cases from Willetts et al. (53) are not included because the number of required treatments is not available.

^b^ n1 is the total number of patients from one technique group, and n2 is the number of successful cases from the same technique group.

### Complications and mortality rate of endoscopic interventions

As shown in Supplementary Table S1 and Table 1, in the endotracheal stent placement group, there was an increase in secretion in the airway of one patient and one patient developed mild esophageal stenosis after the procedure; however, after the stents were removed, both symptoms disappeared. One patient in the de-epithelialization group developed an extensive necrosis of the posterior tracheal wall during the postoperative period, and the airway developed grade II stenosis during the follow-up period. There were no intraoperative or postoperative complications in the chemical sealant–only group. Six patients in the de-epithelialization-chemical sealant group developed an esophageal stricture, but it disappeared at the end of the follow-up period.

With regard to mortality, of the 170 children, two (1.2%) died. One died 3 days after endoscopic de-epithelialization combined with chemical sealant operation, and another died from postoperative fungal septicemia following the endoscopic insertion of the cardiac Amplatzer septal. According to the profile presented in the literature, both deaths were not related to the endoscopic intervention procedures.

## Discussion

Congenital TEF is a rare congenital anomaly in newborns with an incidence of approximately 1/2,500–1/4,500 (60), and it can present with varying degrees of respiratory distress symptoms, choking and cyanosis on feeding, recurrent lower respiratory tract infection, abdominal distension, and failure to thrive. Although 90% of newborns are diagnosed during the first year of life, the presentation may be more insidious and manifest as recurrent pneumonias in older children (41, 64, 61–62). Acquired TEF is not a common anomaly in children, and it is mainly secondary to benign factors such as accidental swallowing of foreign bodies (button batteries, metal objects, etc.), ingestion of corrosive fluids, and iatrogenic injury after tracheotomy and tracheal intubation (9, 10). Given the interference of feeding and a possible fatal pulmonary adverse event of TEF, once detected, a prompt closure of TEFs is critical. Although open surgery or thoracoscopic surgery for TEFs has seen tremendous development over the last few years, recurrent TEFs remain a therapeutic challenge, with the literature reporting up to 20% of recurrence cases (9, 11, 12).

Both recurrent TEF and acquired TEF are anatomically similar to that of congenital H-type TEF. However, both are more challenging to treat than congenital TEF because of postoperative adhesions, sustained chemical damage, inflammation of tissues, scars, and associated complications (63). In the last few decades, endoscopic interventions have emerged as a minimally invasive alternative to the standard open closure procedure. It remains controversial whether endoscopic interventions are better than open surgery for pediatricians and which endoscopic intervention technique is a preferred choice. Therefore, this review aims to provide a systematic analysis to update and summarize the characteristics of the endoscopic interventional treatment method to guide future clinical work in children with TEF.

It is well known that endoscopic intervention is an attractive option because of its high success, low mortality, and low recurrence rates. Previous reviews (38, 64, 65) showed that the overall success rate of endoscopic interventional treatment was 74%–84%. In the present review (Supplementary Table S1), a total of 46 studies describing 170 TEF patients in an age range of 0.3–175 months were included, and the patient breakup is 11 with acquired TEF, 144 with recurrent TEF, and 15 with congenital TEF (H-type). A total of 119 TEFs were successfully blocked through endoscopic interventions with an overall success rate of 70%, which was relatively lower than that of the previous study (65). This could be attributed to the use of new techniques such as tracheal stenting in children, and newly developed techniques without any amendments usually have a high possibility of failure. Regular use and appropriate adjustments can increase the success rate of these new techniques. It is well known that more complications result from the surgical correction of TEF, which include injury to the recurrent laryngeal nerve, secondary vocal cord paralysis, longer tracheal intubation, recurrent fistula, anastomotic leaks, tracheal obstruction, and pneumothorax. However, endoscopic interventions are to be done only through the endoscopic catheter on the mucosa of the fistula, the treatment area of these interventions is limited, and the above surgical complications can be completely avoided (57). As seen in Supplementary Table S1, no obviously severe intraoperative/postoperative complications occurred in patients during the follow-up period. Only a mild esophageal stricture was reported in six patients, grade II tracheal stenosis in one patient, and two died from causes unrelated to endoscopic procedures with a mortality rate of approximately 1.2%, which is much lower than that of open surgery.

There are various endoscopic intervention techniques for TEF in children, such as endotracheal stenting, de-epithelialization alone, chemical sealant alone, and the combination of both. De-epithelialization is a very common procedure in the repair of TEF in children, with low trauma and high treatment success rates. It mainly includes endoscopic diathermy coagulation (EDC), laser (KTP, Thulium, Nd:YAG, Holmium), or argon plasma coagulation (APC) probe to cauterization, mechanical abrasion (bronchial brush, biopsy forceps), and chemical abrasion (silver nitrate, 50% TCA). EDC, laser, and APC are used to close the fistula with the mechanism of cauterization of the fistula mucosa to form a scar tissue in the form of heat and light (65). Endoscopic de-epithelialization is technically easier to perform, is less dangerous, and helps avoid the risk of injury to other important structures and should be used preferentially in the pediatric population. However, we also need to be aware of the fact that endoscopic de-epithelialization also has potentially life-threatening complications. In this review, respiratory distress has been reported in patients following diathermy, which is thought to be an edema of the posterior tracheal wall due to a slightly longer burning time (52). One patient has been reported to have had an extensive necrosis of the posterior tracheal wall that occurred after the application of laser de-epithelialization and ultimately leading to airway stenosis (17). The treatment of this condition requires a great amount of surgical experience, and it is suggested that all surgeons should actively improve their skills to prevent the occurrence of serious complications when performing this treatment technique in the future. In Table 3, we comparatively analyze the success rates of different de-epithelialization techniques and find no significant difference across techniques (*P* > 0.05). Thus, a definitive conclusion on which type of de-epithelialization is better cannot be reached. However, the electrode cautery, unlike the laser, cannot calculate cautery energy, and therefore, the cautery site needs to be carefully observed during the process of cautery to prevent the occurrence of tracheal edema mentioned above. Possibly, even mucosal necrosis and perforation of the local fistula mucosa due to longer cautery times (27) will occur. Nevertheless, in our opinion, even though the energy of the laser is controllable, we cannot ignore its potential risks during the operation, such as the possibility of airway mucosal necrosis resulting from the application of the laser.

**Table 3:** Summary of results comparing different de-epithelialization techniques.

Descriptive data	Chemocauterization (50% TCA, silver nitrate)	Laser	Electrocautery	*χ* ^2^	*P*-value
Number of patients	25	27	12		
Treatment failures converted to open surgery, *n* (%)	5 (20.0%)	10 (37.0%)	3 (25.0%)	1.935	0.413
Successful rate, *n* (%)	20 (80.0%)	17 (63.0%)	9 (75.0%)		

APC, argon plasma coagulation.

Note: When multiple techniques were used on one patient, the last technique described was used to deduce the final outcome.

APC is used, combined with other techniques, so it is not listed here.

Chemical sealant therapy for TEF, as its name implies, is a kind of technique that seals off the fistula by a sealant. Various chemical sealants such as fibrin glue (FG), histoacryl (n-butyl-z-cyanoacrylate) or histoacryl blended with lipiodol, and Glubran2 (cyan acrylic glue) can be used for injection into the fistula. The most common sealant is FG, which consists of a fibrinogen/FXIII concentrate and thrombin concentrated from human plasma. It can form a fibrin-cured substance for repairing hemostasis and tissue adhesion, thereby further sealing the fistula. The FG is more effective in primary wounds where the wound surface is tightly adherent and not fully epithelialized, and where fibroblasts can easily proliferate into the thin layers of the structured fibrin network, eventually forming a durable cross-link (50). Therefore, the use of histoacryl, histoacryl and lipiodol, deflux [a biocompatible dextranomer/hyaluronic acid (Dx/HA) copolymer], and Glubran2 of the fibrin sealant has been reported in the literature. The potential mechanism of action of the combination of these substances is to induce an inflammatory response to trigger fibrosis proliferation to produce a sealing effect (16). The success rate of endoscopic closure of fistulas with chemical sealants alone in this review was approximately only 63.6% (Table 1), and it has been reported that the overall success rate of FG alone is 67% and that of histoacryl alone is 62% (65). The relatively low success rate, compared with other endoscopic interventional techniques, may be attributed to the insufficient inflammation induced by the sealant itself and the inconspicuous proliferation of fibrous cells in the mucosa of the fistula, because of which the fistula cannot be closed. In Table 4, it can be seen that the overall success rate of FG alone is 73.9% and that of histoacryl alone is 44.4%, and there is no statistical significance in the success rate between the two sealant groups, which is consistent with that of the previous report. Therefore, we cannot arrive at a definitive conclusion on which chemical sealant technique is more efficient than others.

**Table 4:** Summary of results comparing different sealant techniques.

Descriptive data	Fibrin glue	Histoacryl	*P*-value
Number of patients	23	9	
Treatment failures converted to open surgery, *n* (%)	6 (23.1%)	5 (55.6%)	0.213
Successful rate, *n* (%)	17 (73.9%)	4 (44.4%)	

Note: When multiple techniques were used on one patient, the last technique described was used to deduce the final outcome.

FG is used, combined with histoacryl in one patient, so it is not listed here.

The principle of sealing fistulas for the combination therapy is basically similar, as they generally first undergo de-epithelialization to induce mucosal inflammation, which subsequently reacts with the sealant injected into the fistula to promote mucosal fibrosis and granulation tissue proliferation to seal the fistula. For chemical sealants, adequate de-epithelialization and induction of mucosal inflammation are essential to improve the coagulation and sealing effect of chemical sealants. For example, fibrin glue alone does not trigger sufficient inflammation to fibrosis, which requires de-epithelialization of the fistula. There are various treatment combinations such as mechanical injury/laser/thermal therapy for de-epithelialization, followed by fibrin glue and histoacryl injection into the fistula, which accelerate the coagulation of the chemical sealant through the local inflammatory response after de-epithelialization of the fistula mucosa to achieve the final closure of the fistula. In addition, sclerosing agents such as DuraSeal, Dx/HA, or aethoxysklerol are injected into the submucosa of the fistula and these can also induce swelling and inflammatory responses in the fistula mucosa, leading to de-epithelialization and sealing of the fistula when combined with a sealant (56, 58). The potential inflammation-inducing properties of synthetic polymeric tissue adhesive sealants may theoretically make them superior to FG in terms of sealing effectiveness, but this has not been accurately verified. Taking the above mechanism into consideration, the combination of de-epithelialization with chemosealant occlusion therapy will theoretically improve the sealing effect in TEF. According to the statistical result of this review (Table 1), the success rate of the combined therapy is 77.4%, which is higher than that of either de-epithelialization alone (72.3%) or sealant alone (63.6%), and is consistent with that of previous literature (38). There are numerous reports on endoscopic de-epithelialization in random combination with sealant therapy for TEF. No prospective and multisample studies have been reported to date to prove which combination is the optimal treatment, and this research gap needs to be bridged in the future. However, the results of our review indicate a good application prospect for combined endoscopic treatment to seal the fistula. In addition, two patients in this group were treated with combined therapy with the addition of a biosynthetic mesh, and it successfully blocked the fistula (31, 39). The literature reports that the biosynthetic mesh provides a durable and stable collagen lattice that allows the binding of fibroblasts and capillaries to form scars that keep growing and that the biosynthetic scaffold degrades over time, but the tissue that replaces it remodels itself more strongly than natural tissue (39).

Coated endotracheal metallic stents have been widely used in adults to treat TEFs with satisfactory results but have been found not suitable for surgery, whereas endotracheal stenting in children is a novel attempt, because the coated respiratory metallic stents meant specifically for children are not presently available. In light of this, coated metallic biliary stents (15, 20, 23, 30) woven from a nitinol alloy wire and coated on the surface are becoming increasingly popular with the pediatrician because of its features of stronger adhesion and a close fit with the trachea compared with silicone stents. In this review (Table 1), only one patient was treated with a silicone stent; however, the treatment resulted in failure, while the remaining five patients were treated with fully covered endotracheal metallic biliary stents. Of all the six patients in whom endotracheal stenting was used, one case of a patient with acquired TEF was successfully sealed with an overall success rate of 16.7%. In accord with the literature, endotracheal stenting placement for TEF is commonly used for acquired TEF, especially in those children who accidentally swallowed a button battery. It is well known that the corrosive damage secondary to battery ingestion is caused by the toxic effect of mercuric oxide, by the mucosal burning effect of the local discharge of battery contents, and additionally by local necrosis due to direct pressure, all of which can often result in a large tracheoesophageal fistula orifice that is difficult to occlude through conventional surgical treatment and other types of endoscopic repair (66, 67). Because of its easy adjustability, the coated biliary stent is a good fit for repairing this type of TEF, and therefore, it is clinically feasible to consider endotracheal stenting if the fistula area is large. In addition, it has been reported that some acquired TEFs have the tendency to self-heal (9). So, some children with small fistulas can be temporarily observed for self-healing in the absence of obvious symptoms, and if there is no tendency for self-healing and even clinical symptoms worsen, the endotracheal stent can be promptly applied to achieve fistula closure. It should be noted that endotracheal stenting may result in complications such as chest pain, gastrointestinal bleeding, stent food impaction, stent displacement, and secondary fistula. In this review, none of these complications occurred in patients after airway stent placement, and only tracheal secretions increased in one patient, while a mild stricture occurred in another (15, 20), which may be attributed to the limited number of cases.

There are also some unusual methods to seal TEF, such as endoscopic submucosal dissection and insertion of the cardiac Amplatzer septa and the flexible endoscopic suturing device (preloaded suture device + Ti-knot device) (21, 28, 40). Two acquired TEFs failed to close with the insertion of the cardiac Amplatzer septal, one patient was kept stable for 4 years, but finally the treatment was converted to surgery, and another patient died after being critically ill with fungal septicemia (28). Fortunately, one acquired TEF was successfully closed by endoscopic submucosal dissection combined with endoscopic clips, and one recurrent TEF was successfully closed with the flexible endoscopic suturing device. These techniques have been less performed in medical institutions, and the overall treatment outcome and success rate of the intervention techniques cannot be predicted because of the limited number of cases. But these methods are innovative and may be promising for the future, and hopefully, these could be improved and used in more children with TEF.

According to the literature, the success rate of de-epithelial treatment was 62.5%–87%, the success rate of chemical sealant was 78.6%–83.4%, and that of the combination method was 83.4%–93.3%, and no significant difference in the success rate across these techniques has been reported (64). According to the statistical result of this review, the success rate of the de-epithelialization technique is approximately 72.3%. The success rate of the chemical sealant–alone technique is approximately only 63.6% and that of the de-epithelialization and chemical sealant combination therapy is 77.4%, which are consistent with that of previous literature (38) and previous reviews (38, 65). To date, there have been no reports about the success rate of the endotracheal stenting technique in children with TEF. So, it is for the first time that we performed a review and summarized the published literature about the endoscopic tracheal stenting technique in children with TEF, and its success rate in our review is 16.7%. It was reported that in a previous adult stenting treatment for TEF, the stent completely sealed off the fistula in 49 of 61 patients with an overall success rate of 80% (68). In contrast, the reported success rate of endotracheal stenting therapy in adults is significantly higher than that in this review, which might be closely related to the small sample size of children who underwent stent therapy for TEF in our systematic review. It is also because the successful cases of adults included the cases sealed off by keeping the stent into the trachea permanently, unlike the cases of children. The successful occlusion of TEF was defined as a complete repair of the fistula after removing the stent and not just sealing it off with endotracheal stents. Although in this review, the failure rate after stenting is high, the acute clinical symptoms of children after stenting are alleviated, and the fistula area is reduced, which establishes the foundation for later surgical closure. Shown in Table 1 is a comparison of the success rates across multiple endoscopic interventional technique groups that showed a significant difference with *P *< 0.05 (*P *= 0.024), and the technique of the combination of de-epithelialization and sealant seems to have the highest success rate (77.4%). However, we cannot arrive at a definite conclusion to the effect that the combination of the de-epithelialization and sealant techniques is the first option for children, because the group-to-group comparison of these different techniques did not show any significant difference (*P *> 0.05), as also the comparison of the stenting group and the combination of the de-epithelialization and sealant groups (*P *< 0.05). Therefore, more endoscopic interventions for TEF in children are required to reach a definite conclusion.

Pediatricians are always curious to know for what type of TEF endoscopic interventional treatment is more suitable. In this review, as shown in Table 2, 103 out of 144 acquired TEFs were successfully blocked with a success rate of 71.5%, 9 out of 15 cases (60%) of congenital TEF were successfully obliterated, and 7 of 11 cases (63.6%) of acquired TEF were successfully blocked. The comparative analysis of the success rate showed that there was no significant difference across different TEF-type groups (*P *> 0.05), which may be related to the limited number of cases included in different TEF-type groups, warranting more endoscopic interventions in the future. According to the literature, the result of endoscopic de-epithelialization treatment in congenital THE (H-type) is superior to that in recurrent fistulae because of the inclination of the canal, which allows better wall jointing after disruption of the mucosa and contributes to fistula occlusion. However, the fistulas in recurrent TEF cases are short and have direct connections, and therefore, more treatment sessions are required to allow a gradual healing of the fistula before complete occlusion (52). As shown in Table 3, 9 out of 15 congenital TEFs (60%) were successfully occluded by de-epithelialization (alone and combination with sealant), while 81 out of 107 recurrent TEFs (75.7%) were completely occluded. Even though there is no statistical significance, it appears that recurrent TEFs show better treatment results than congenital TEFs, and more endoscopic interventions are necessary to validate this point in the future.

Apart from the minimally invasive, safe, and high success rate characteristics of endoscopic interventional techniques, another significant characteristic is repeatable, and many patients with TEF usually need more than one endoscopic intervention. All endoscopic interventional techniques can be operated repeatedly, as indicated by Table 1. The mean number of treatments required for successful closure of TEF by all interventional techniques, except stenting, is more than 1 time. In this systematic review, only one patient was successfully occluded by stenting with a one-time treatment. However, it is not convincing to argue that only one procedure is required just because only one attempt was made in all stenting cases. As indicated by Table 2, more than one procedure was needed to successfully occlude different types of TEF, and there was no significant difference among the success rates in groups of different types.

### Limitations

1. The literature included in this review was limited to the English language in the exclusion criteria, which may lead to an inadequate final inclusion and affect the statistical result.
2. In this review, 37 papers out the 46 included from the literature are case reports or very small personal series (*n* < 6), which reflects that many endoscopic treatment techniques are still in the exploratory stage. Although they have achieved a good blocking effect, large samples and prospective studies are needed to validate them in the future. In addition, 24 papers were published before 2010, which may affect the effectiveness of different interventional techniques in actual terms.
3. In this review, de-epithelialization, chemical sealant blocking, and the combination of both included a variety of treatment techniques. There is no solid evidence to prove which endoscopic interventional technique is the best for TEF, and this is an important gap that needs to be filled. Such evidence would be very meaningful for the future endoscopic treatment of TEF in children.

## Conclusion

In conclusion, the original purpose of both surgical procedures and endoscopic interventional therapies is to close the fistula successfully, while reducing recurrence and complications as much as possible and further obtain the best results with minimal trauma. With the development of interventional pulmonology, numerous endoscopic techniques offer an alternative to occlude the fistula in a large number of children with TEF because of their safety, minimally invasive feature, and high success rate. However, even though the existing endoscopic techniques have achieved good results, especially the combination therapy of de-epithelialization and chemical sealant, there are still limitations and risks associated with endoscopic treatment. Therefore, we need to explore new techniques or continue to implement and optimize the existing ones in the future.

## Data Availability

The original contributions presented in the study are included in the article/Supplementary Material, further inquiries can be directed to the corresponding authors.
