# Supplementary material for: Endoscopic interventional therapies for tracheoesophageal fistulas in children: A systematic review
Source: Front Pediatr. 2023 Feb 22;11:1121803. doi: 10.3389/fped.2023.1121803 (PMC9992425; doi:10.3389/fped.2023.1121803)
Supplement: Supplementary file 1 [file Table1.docx]

**Table S1**. Summary of published endoscopic treatments of tracheoesophageal fistula

| Reference | specialty of the authors | Numbers of patients | Mean age at repair in mo M/m(range)^a^ | Mean number of treatments required^b^ | Treatment failures converted to surgery n | Mean length of follow-up in mo M/m (range)^c^ | Endoscopic repair successful rate n (%) | Endoscopic techniques | Intraoperative/Postoperative complications | TEF-type^d^ | Preoperative symptom | Notes |
| --- | --- | --- | --- | --- | --- | --- | --- | --- | --- | --- | --- | --- |
| Ferreira Silva J, etc. 2022(15) | Gastroenterology | 1 | 24 | - | 1 | 4 | 0 | Fully-covered metallic biliary stent placement with modified shim | Increase of tracheal secretions | A (button-battery ingestion) | Oral intolerance, respiratory distress |  |
| Sautin A, etc. 2021(16) | Pediatric Surgery | 11 | M21(1.2-175) | 2.25 | 3 | M44.5(15.6-62.4) | 8(72.7%) | De-epithelialization (laser) | No | R |  |  |
| Luscan R, etc. 2021(17) | Pediatric Otolaryngology | 11 | m19(1.1-63） | 1 | 7 | M24(14-72) | 4(36.4%) | De-epithelialize (Thulium laser) in 10pts, De-epithelialization (Thulium laser) +FG in 1pt | Tracheal posterior membrane necrosis with finally grade II tracheal stenosis in 1pt | 6R+5C(H-type) | Chronic coughing, RP, cyanosis and aspiration on feeding, recurrent wheezing |  |
| Miro I, etc. 2020(18) | Pediatric Surgery | 14 | m2.8(0.5-25.7) | 2.6 | 4 | m145（120-240） | 10(71.4%) | De-epithelization (EDC: metallic guide 3F pediatric urethral stent) + FG in 11 pts; FG in 3 pts | NO | R | - | 8 of 11pts succeeded by de-epithelization+ FG and 2 of 3pts succeeded by FG alone |
| Valiyev N, etc. 2019(19) | Pediatric Surgery | 9 | M18(4-60) | 1 | 8 | M28(3-60) | 1(11.1%) | De-epithelization (laser) + FG in 4pts; De-epithelization (50%TCA) in 4pts; De-epithelization (50%TCA+lacer) +FG in 1pt | No | R |  | Successfully obliterated in 1pt with de-epithelization (laser) + FG |
| Zhongxiao Z, etc. 2017(20) | Respiratory Intervention | 1 | 10 | - | 1 | 25 | 0 | Fully-covered metallic biliary stent | Mild ES | A (button-battery ingestion) |  |  |
| Nazir Z, etc. 2017(9) | Pediatric Surgery | 5 | M9(3-27) | 2 | 0 | M36(7-36) | 5(100%) | De-epithelization (Bugbee electrocautery) + FG | ES required dilatation in 3pts | 2A (Ballpoint pen, Button battery ingestion) + 3R | Choking on feeding, recurring respiratory infections |  |
| Gruner M, etc. 2017(21) | Endoscopy and Gastroenterology | 1 | 36 | 4 | 0 | 3 | 1(100%) | Failed in twice attempts of covered esophageal stenting and once De-epithelialization (APC); then succeeded by ESD + clips | No | A (button-battery ingestion) | - |  |
| Gregory S, etc. 2017(22) | Otolaryngology | 1 | 8 | 1 | 0 | 5 | 1(100%) | De-epithelialization (APC + Bugbee electrocautery) | No | R | Recurrent coughing，frequent respiratory illnesses |  |
| Wang Lina, etc. 2016(23) | Pediatric Respiration | 3 | M20（3-29） | 1 | 2 | 2-12 | 1(33.3%) | Fully-covered metallic biliary stent in 2pts; silicone stent in 1pt | No | 2R+1A (button battery ingestion) |  | Successfully closed in 1pt with metallic biliary stent |
| Maizlin II, etc. 2016(24) | Pediatric Surgery and Otolaryngology | 1 | 108 | 1 | 0 | 1 | 1(100%) | De-epithelialization (Bugbee electrode) + FG | ES | A (gunshot wound) | Abscess formed in the anterior neck |  |
| Lelonge Y, etc. 2016(25) | Pediatric Surgery and Urology | 14 | M7.5(3-156) | 1.9 | 0 | m41(8-72) | 14(100%) | De-epithelialization (50 % TCA) | No | 2C(H-type) +12R | Coughing, pneumonia |  |
| Cadena-León JF, etc. 2016(26) | Gastroenterology | 1 | 36 | 1 | 0 | 24 | 1(100%) | Histoacryl and lipiodol | No | R | RP, GER, bronchial hyperreactivity, esophageal candidiasis, pediatric chronic lung disease, and chronic malnutrition |  |
| Victoria VL, etc. 2015(27) | Surgery | 1 | 33 | 1 | 0 | 18 | 1(100%) | De-epithelization (electrocautery) + FG | No | A (Foreign body ingestion) |  |  |
| Cohen Atsmoni S, etc. 2015(28) | Otolaryngology-Head and Neck Surgery | 2 | - | - | 1 | 0.5-48 | 0 | Insertion of cardiac Amplatzer septal occulder through trachea in 1pt, but through esophagus in another 1pt. | No | A (prolonged mechanical ventilation) |  | 1pt kept stable for four years; another 1pt died of fungal septicemia |
| Benatta MA, etc. 2014(30) | Digestive Endoscopy Unit | 1 | 2 | - | 0 | 12 | 0 | Partially-covered metal biliary stent | No | R | Cyanosis on feeding and recurrent lung infections with body weight loss | Ten weeks after the stenting, the fistula kept existing while finally obliterated by several endoclips |
| Propst EJ, etc. 2014(29) | Otolaryngology-Head and Neck Surgery | 1 | 4 | 2 | 0 | 12 | 1(100%) | Failed in de-epithelialization (Bugbee electrocautery) +FG; succeeded in De-epithelialization (Bugbee electrocautery) + endoclips | No | R | Aspiration pneumonia, choking on feeding |  |
| Van Niekerk ML. 2012(31) | Pediatric Surgery | 1 | 6 | 1 | 0 | 24 | 1(100%) | De-epithelization (brush)+ Permacol (porcine biological mesh) held in place by DuraSeal | No | R |  |  |
| Nardo GD, etc. 2012(32) | Gastroenterology | 1 | 2 | 2 | 0 | 24 | 1 (100%) | Glubran2 injection showed a caliber reduction, De-epithelization (APC) got complete closure | No | C | Persistent coughing during breastfeeding，regurgitation |  |
| Briganti V, etc. 2011(33) | Pediatric Surgery | 5 | M8(1-18) | 1 | 3 | - | 2(40%) | De-epithelization (brush and/or biopsy forceps) + Deflux injected into the submucosa | No | R | RP, cough, apnea on feeding, |  |
| Rakoczy G, etc. 2010(34) | Pediatric Surgery | 1 | 3 | 3 | 0 | 24 | 1(100%) | Failed in twice attempts (Electrocautery+ injection of FG); De-epithelialization (KTP laser) got final success. | No | R | GER，dysphagia |  |
| Josefina F, etc. 2010(35) | Pediatric Surgery | 1 | 0.6 | 1 | 0 | 22 | 1(100%) | FG injection into the submucosa | No | R | Severe respiratory distress, progressive dyspnea, |  |
| Yoon JH, etc. 2009(36) | Surgery and Gastrointestinal Endoscopy | 1 | 156 | 1 | 0 | 8 | 1(100%) | De-epithelialization (brush) + (Histoacryl +lipiodol) | No | R | Choking with oral feeding, and respiratory symptoms |  |
| Richter GT, etc. 2008(38) | Otolaryngology-Head and Neck Surgery | 4 | M11.5(3-20) | 1.25 | 0 | m19.5(4-38) | 4(100%) | De-epithelialization (Bugbee electrocautery) + FG | ES in 3pts | R | Recurrent wheezing，coughing，cyanosis on feeding，persistent pneumonia |  |
| Keckler SJ, etc. 2008(39) | Surgery | 1 | 12 | 3 | 0 | 36 | 1 (100%) | Failed in de-epithelization (brush)+ Biosynthetic mesh + FG at the first attempt, subsequently succeeded in twice attempts of placing mesh into the tract | No | R | Pneumonia，coughing after meals |  |
| Sung MW, etc. 2008(37) | Otorhinolaryngology | 3 | M16(1-156) | 3.3 | 0 | m14(9-22) | 3(100%) | De-epithelialization (50 % TCA) | No | R | Recurrent chronic pulmonary disease |  |
| Rocca R, etc. 2007(40) | Gastroenterology | 1 | 72 | 4 | 0 | 22 | 1(100%) | Two attempts failed (Endoscopic abrasion of the mucosa surrounding the orifice+ metallic clips application); Glubran2 failed too; finally succeeded by flexible endoscopic suturing device (preloaded suture device+ Ti-knot device). | No | R | Persistent cough; RP |  |
| Meier JD, etc. 2007(41) | Otolaryngology-Head and Neck Surgery | 3 | M5(5-108） | 2 | 1 | 36-60 | 2(66.7%) | De-epithelization (brush)+FG | No | R | Respiratory distress, coughing and choking on feedings |  |
| Tzifa KT, etc. 2006(42) | Otolaryngology-Head and Neck Surgery | 10 | M10(0.3-84) | 1.5 | 1 | M36(3-108) | 8(80%) | De-epithelialization (Bugbee electrocautery or mechanical abrasion) + (Histoacryl and lipiodol) | No | 7R+2C(H-type) +1A(Traumatic) |  | 1pt died three days after the procedure due to causes unrelated to the operation |
| Gutierrez SRC, etc. 2006(45) | Pediatric Surgery | 7 | m0.56(0.47-0.67) | 1.5 | 1 | m88.8(24-132) | 6(85.7%) | De-epithelization (EDC: metallic guide 3F pediatric urethral stent) + FG in 4 pts; FG in 3 pts | No | R |  | 3-sessions of FG injection failed in 1pt and converted to open surgery |
| Linder Arne. 2006(43) | Otolaryngology-Head and Neck Surgery | 4 | m82.5(12-156) | 4.3 | 1 | m45(24-72) | 3(75%) | De-epithelialization (Using silver nitrate to denude) | No | R | Airway symptoms |  |
| Ishman SL, etc. 2006(44) | Otolaryngology | 1 | 18 | 2 | 0 | 18 | 1(100%) | Failed in de-epithelization(laser) alone; succeeded by de-epithelization (KTP laser) + FG. | No | R | RP |  |
| Khurana S, etc. 2004(47) | Pediatric Surgery | 6 | M7(0.5-54) | 2.2 | 1 | m53(16-96) | 5(83.3%) | De-epithelialization (EDC: metallic guide 3F pediatric urethral stent) | No | R | Choking and spluttering on feedings |  |
| Ogunmola N, etc. 2004(46) | Gastroenterology | 1 | 60 | 1 | 0 | 12 | 1 (100%) | FG | No | R | RP, recurrent reactive airway disease |  |
| Lopes MF, etc. 2003(48) | Pediatric Surgery | 1 | 11 | 2 | 0 | 36 | 1(100%) | Failed in FG injection; succeeded by injection of Histoacryl and polidocanol into the submucosa via esophagoscopy | No | R | - |  |
| McGahren ED, etc. 2001(49) | Pediatric Surgery | 1 | 3 | 3 | 0 | 42 | 1(100%) | De-epithelialization (using wire to cauterize) + FG | No | R | Coughing on feeding |  |
| Ng WT, etc. 1999(50) | Surgery | 1 | 1 | 3 | 0 | 24 | 1(100%) | Two failed attempts with FG; succeeded at third attempt with De-epithelialization (holmium laser) + FG | No | R |  |  |
| Bhatnagar V, etc. 1999(52) | Pediatric Surgery | 5 | M5(0.5-156) | 2.3 | 2 | 3 | 3(60%) | De-epithelialization (EDC: insulated diathermy wire) in 3pts; De-epithelialization (Nd:YAG Laser) in 2pts | Respiratory distress (3 pts with diathermy) | 2C(H-type) +3R | Choking, coughing, cyanosis on feeding, RP | Endoscopic diathermy failed in 2pts and converted to open surgery |
| Hoelzer DJ, etc. 1999(51) | Pediatric Surgery | 1 | 40 | 1 | 0 | 48 | 1(100%) | FG | No | R | Asthma, pneumonia |  |
| Willetts IE, etc. 1998(53) | Pediatric Surgery | 22 | 1-144 | - | 10 | 3-264 | 12(54.5%) | Histoacryl in 8pts; FG in 11pts; Histoacryl + Aethoxysclerol in 2pts; Histoacryl +FG in 1pt | No | R |  | 3 of 8 pts succeeded with Histoacryl; 7 of 11pts succeeded with FG alone; 2pts succeeded with Histoacryl + Aethoxysclerol; 1pt failed with Histoacryl and FG in combination. |
| Wiseman NE, etc. 1995(54) | Pediatric Surgery | 2 | 2.25-45 | 2.5 | 0 | 6(only case 2) | 2(100%) | FG | No | R | Coughing, RP, difficulty in feeding | Endoscopically using electrocautery and Histoacryl / thoracotomy failed in 1pt; converted to open surgery, but still not obliterated |
| Gutierrez C, etc. 1994(55) | Pediatric Surgery | 1 | 0.5 | 1 | 0 | 3 | 1 (100%) | FG | No | R | Respiratory distress syndrome |  |
| Vandenplas Y, etc. 1993(56) | Gastroenterology | 1 | 144 | 4 | 0 | 12 | 1(100%) | Failed in three attempts (Injecting FG, Histoacryl, Histoacryl+ submucosal injection of 2ml 30%NaCl); succeeded by De-epithelialization (Aethoxysclerol inject in the submucosa) + Histoacyl | No | R | Nocturnal coughing；ingestion-related coughing |  |
| Schmittenbecher PP, etc. 1992(57) | Pediatric Surgery | 3 | M1(1-1.5) | 1 | 1 | 3 | 2(66.7%) | De-epithelialization (Nd: YAG Laser) | No | C | Apnea, choking, coughing with feeding, respiratory distress, cyanosis on feeding |  |
| Al-Samarrai AY, etc. 1987(58) | Pediatric Surgery and Gastroenterology Surgery | 1 | 3 | 1 | 0 | 12 | 1(100%) | Histoarcyl + De-epithelialization (0.5% Polidocanol inject into submucosa) | No | R | Coughing, dyspnea, frothy sputum |  |
| Rangecroft L, etc. 1984(59) | Pediatric Surgery | 2 | 60(case 1) | 5.5 | 0 | 12 | 2(100%) | De-epithelialization (EDC: insulated diathermy wire) | No | R | Eructing large amounts of gas from the stomach/severe aspiration pneumonia |  |
| Total/range 46 |  | 170 | 0.3-175 | - | 48(28.2%) | 0.5-264 | 119(70.0%) | - | - | 144R+15C+11A |  | 2pts died |

**Abbreviations**: TEF: tracheoesophageal fistula; FG: fibrin glue; TCA: Trichloroacetic acid; APC: argon plasma coagulation; ESD: endoscopic submucosal dissection; KTP: Potassium titanyl phosphate laser; ES: esophageal stricture; RP: recurrent pneumonia; GER: gastroesophageal reflux; EDC: endoscopic diathermy coagulation; Nd:YAG: neo-dymium: yttrium-aluminum-garnet laser; KTP laser: potassium titanyl phosphate laser; n: number; M: median; m: mean; y: year; mo: month; w: week; d: day; pt: patient; OS: open surgery.

Note: Permacol: porcine dermal biological mesh; Deflux: a biocompatible dextranomer/hyaluronic acid (Dx/HA) copolymer; Histoacryl: n-butyl-z-cyanoacrylate; Polidocanol: aethoxysclerol; Glubran2: cyan acrylic glue.

^a c^M/m stands for Median/mean

^b^Mean number of successful treatments required for successfully treated cases.

^d^TEF type: A-Acquired TEF, R-Recurrent TEF, C-Congenital TEF
